# THE STRUCTURAL AND COGNITIVE SOCIAL CAPITAL OF KOREAN OLDER ADULTS AND THEIR DEPRESSION TRAJECTORIES

**DOI:** 10.1093/geroni/igac059.2636

**Published:** 2022-12-20

**Authors:** Jinhyun Kim, Sun-Bi Um, Xiang Shi, Hyunjung Park

**Affiliations:** Pusan National University, Geumjeong-gu, Pusan-jikhalsi, Republic of Korea; Pusan National University, Geumjeong-gu, Pusan-jikhalsi, Republic of Korea; Pusan National University, Geumjeong-gu, Pusan-jikhalsi, Republic of Korea; Pusan National University, Geumjeong-gu, Pusan-jikhalsi, Republic of Korea

## Abstract

Despite the rapid increase of older population in South Korea, we do not understand the patterns of their social capital and relationships with depression trajectories in depth. This study aims to identify the patterns of social capital of Korean older adults aged 65 years and older based on latent class analyses and their depression trajectories by latent classes. For data analyses, 3,606 Korean older adults from the Korean welfare panel study data were selected. Latent class analyses were conducted by using various components of structural social capital such as number of contacts with family, friends, and neighbors and cognitive social capital such as trust, reciprocity, and satisfaction from social relationships. Results showed that three latent classes were found. We named the first class as a strong structural and cognitive group because it showed the highest levels of number of social contacts, trust and reciprocity. The second class was called as a weak structural and strong cognitive group because it showed lower levels of social contacts but higher levels of satisfaction from social relationships. The third class was called a weak structural and cognitive group because it showed the lowest levels of social contacts, trust, reciprocity, and satisfaction from social relationships. Results from latent growth curve modeling, the second class showed the lowest depressive symptoms but the third class showed the highest depressive symptoms at baseline. Only the third class showed significant rate of changes in depression. Differences in depression trajectories by three latent classes need to be further discussed.

